# Massage in Rheumatism of the Temporo-Maxillary Articulation and Muscles of the Lower Jaw

**Published:** 1894-08

**Authors:** Douglas Graham

**Affiliations:** Boston, Mass.


### MASSAGE IN RHEUMATISM OF THE TEMPORO-MAXILLARY ARTICULATION AND MUSCLES OF THE LOWER JAW.[1]

BY DOUGLAS GRAHAM, M.D., BOSTON, MASS.

On January 7, 1891, there came to my office a young lady, nineteen years of age, who was naturally slim, delicate, and loose-jointed. She had difficulty in chewing, difficulty in articulating, and, on examination, it was found that she could open her mouth but three-eighths of an inch between her front teeth, and that the lower jaw receded behind the upper three-eighths of an inch. The muscles that elevate the lower jaw were rigid and stiff, and on account of the difficulty in chewing, her diet was mainly liquid. Attempts at pulling the jaw downward and forward caused pain. This state of affairs had existed for about ten days, and for eleven days before this she had been much worse. By dint of cross-questioning I learned that three weeks before she came to me she woke up frequently at night clinching her teeth and dreading to move her jaw, and that for four or five days afterwards she was feverish, had bad breath and sour-smelling perspiration; from which I inferred that she most likely had an attack of acute articular rheumatism, in which the muscles and articulations of the lower jaw alone were affected.

After thirty minutes of massage to the muscles of the lower jaw, inside and out, alternating with careful, forcible depression and forward traction, the mouth could be opened voluntarily farther and easier, much to the delight of the patient. The following day she reported that she could chew a little better, and had more "catches," as she called them, than she had when she was well. Before massage this day she opened her mouth five-eighths of an inch, a gain of two-eighths over the previous day; and after massage and pulling

---

[1] Read before the American Academy of Dental Science, April 4, 1894.

for twenty or thirty minutes she opened it thirteen-sixteenths of an inch, a further gain of three-sixteenths of an inch; and the lower front teeth were one-fourth of an inch behind the upper, an improvement of one-eighth of an inch in this respect. Next day she opened her mouth thirteen-sixteenths of an inch before massage, fourteen-sixteenths after. Forced opening hurt less than formerly, but as yet there was no perceptible lateral motion, though the muscles felt stronger to her. The day after this she opened the jaw fourteen-sixteenths of an inch before massage, fifteen-sixteenths after; and there was very decided lateral motion to the left, more than to the right. Two days later she opened her mouth fourteen-sixteenths of an inch before massage, and the same after massage and forced depression; and as this did not cause any more discomfort, it seemed as if "we had got to the end of our rope." Hitherto there had been a feeling of lameness for an hour or so after treatment, and when this passed away the muscles and joints felt better than before; and this is usually the way that muscles behave after massage, when suffering from muscular rheumatism. The lower front teeth then closed one-sixteenth of an inch behind the upper, a gain of five-sixteenths of an inch in five days.

At the end of a week the patient reports that she does not get so quickly tired in eating, and that the food does not lodge, nor the jaws "catch," as formerly, even when she considered herself well. Still she knows that the mouth does not yet open as far as it ought to, for she cannot get her tooth-brush "away back." At the end of nine days she can chew well with both sides, and talks almost naturally; and there is no discomfort in forced depression of the lower jaw, which now opens fifteen-sixteenths of an inch voluntarily. January 21, two weeks from the time we began treatment, the jaw slips less than it has for more than a year, and the muscles feel firmer and stronger; opens voluntarily one inch, and lateral motion increases. Five days later opens jaw one and one-eighth inches, chews vigorously, and articulates easily, and apparently is better than she was before she had this trouble. She had eleven massages, with forcible downward traction, and some pulling at home by herself at stated times, as directed.

Six weeks later she came to me again, stating that the jaw had been well until that morning, when, chewing a piece of soft bread, she had an unusually severe "catch," which left an ache and a swollen feeling. Before massage and pulling she opened her mouth on this date one and a half inches, a gain of half an inch since she last visited me; after massage, one and eleven-sixteenths, a gain of three-sixteenths of an inch in about twenty minutes, and the muscles, as she said, "felt real nice and the tied-up feeling gone." She visited me twice more at this time and has since continued well, as I have been informed by her parents. At her last visit to me I advised her not to pull any more at her lower jaw, for I found that one and eleven-sixteenths inches was much more than I could open my own mouth. Of late I have measured the distance between the front teeth of a number of people, and find that the average space they can open their mouths is one and a half inches. In this lax-muscled, loose-jointed individual, it seemed as if we might easily have gone too far in our efforts at making the mouth open wide. And in this connection I could not help recalling the epitaph on a Southern tombstone:

> "Here lies Robert Gordin,
> Mouth almighty and teeth accordin';
> Tread lightly over this great wonder,
> If he opens his jaw, you're gone, by thunder!"

A few words as to diagnosis. The painful cutting of a lower wisdom-tooth is said to sometimes cause a spasmodic contraction of the masseter muscle by contiguous irritation. The spasm is described as being continuous and persistent, not varying in intensity, but a true tonic spasm, the muscles being permanently set so as to keep the jaws nearly closed and susceptible of only slight separation. The pain varies much, being frequently of a dull aching character like rheumatism, for which it may be mistaken, being diffuse and erratic, and may extend up the side of the head and down to the shoulder. I do not think that the case just reported was one of this kind, for other joints had behaved in a similar manner to those of her lower jaw. Prior to two years before I saw her she had had slipping of her hip-joints with pain, often causing her to fall, and this troubled her for a year; and for two years she had snapping and cracking of the joints of her arms and shoulders on putting her arms to her head, and when she came to me there was pain in her left shoulder which could hardly be called anything but rheumatism. Moreover, the physician who attended her during the few days of the acute febrile trouble in connection with her jaws has recently been my patient, and he remembers the case distinctly as one of acute rheumatism of the temporo-maxillary articulation.

With regard to causation, it would seem as if the muscles of this patient's lower jaw acted in a hesitating, stammering, inco-ordinate manner, much in the same way that the muscles of the hand and arm do in some cases of writer's cramp and allied affections, the treatment of which by massage and systematic exercises frequently results favorably. And in loose-jointed individuals it is possible that lax-muscles may allow too much traction upon the ligaments, the irritation of which may beget contraction or contracture of the muscles in order to protect the joint. Slight irritation arising in this or any other way might be sufficient to excite rheumatic arthritis and muscular rheumatism in those predisposed thereto.

The muscles that raise the lower jaw (the temporal masseter and internal pterygoid), as well as those that move it forward (the superficial portion of the masseter, the internal and external pterygoid) and backward (the deep fibres of the masseter and posterior fibres of the temporal) and laterally (the internal pterygoid), seemed to be in a state of subacute myositis or muscular rheumatism, their fibres agglutinated so that they could not glide freely over each other, but contracting partially and irregularly, and pulling unequally, causing unnatural pressure and traction upon terminal nerve-filaments. This condition is quite amenable to massage in other muscles, and probably here also, if care be taken not to attempt too much at one time and thus increase the irritation. As the depressors of the lower jaw (the digastric, the stylo-hyoid, the mylo-hyoid, and the genio-hyoid) must suffer in their circulation and nutrition from inactivity when the mouth cannot be opened, they also received massage. These muscles, together with the temporal and masseter, are the most accessible to external manipulation, while those attached to the inner surface of the lower jaw are very inaccessible and can better be acted upon by passive and active motion. Indeed, the anatomical arrangement of the structures of the temporo-maxillary articulation is not so favorable for massage as is that of the joints of the limbs where we can get all around them.

We are probably safe in presuming that the glenoid cavities in this patient were too large for the condyles of the lower jaw, and that the anterior aspect of these was most likely encroached upon by rheumatic thickening. This was not so easily got at. But a little reflection will convince one that joint-surfaces can be made to exercise an intermittent compression—a sort of massage—upon each other, by means of active and passive motion, whereby the trituration and absorption of deposits in and around them can be hastened.

Cases of rheumatism affecting the temporo-maxillary articulation and muscles of the lower jaw may frequently be seen by gentlemen in the profession of dentistry, but I think they are so rarely met with by others that they are apt to be considered as belonging to some unknown and unclassified list of affections. Professor Andrews has recently sent me a similar, almost a parallel, case to the one just reported, in which no gain in opening the mouth was made until the depressors of the lower jaw had received special attention in the way of massage and resistive motion; that is, opposing contraction or attempting to hold the mouth closed by pushing upward upon the chin while the patient opens the mouth, being careful to keep the resistance less than the strength of the contracting depressors, which requires some practice in order to graduate the resistance instinctively. This case has not been sufficiently long under observation to say much about it.

The effects of massage in such cases would seem to be simultaneously upon the muscles, nerves, and circulation: upon the muscles, separating adhesions between their fibrils so that they can glide freely over each other, thus preventing partial and irregular contractions and removing undue pressure from terminal nerve-filaments; upon the nerves, whereby not only is pressure removed, but a direct sedative effect is produced; upon the circulation, so that it is increased in speed and quantity in the parts *masséed*, thus bringing more nutritive materials and removing more waste products. These influences indirectly affect the joint, which, however, is more acted upon by passive and active motion, which tend to break up deposit, adhesion, and thickening, to spread them over a greater surface, and to propel them onward into the lymph- and blood-currents. Resistive movements train the muscles to act steadily, strongly, and regularly, and thus suppress irregular, spasmodic, and useless movements.

